# Editorial: Advances and challenges to bridge computational intelligence and neuroscience for brain-computer interface

**DOI:** 10.3389/fnrgo.2024.1461494

**Published:** 2024-08-06

**Authors:** Avinash Kumar Singh, Luigi Bianchi, Davide Valeriani, Masaki Nakanishi

**Affiliations:** ^1^School of Computer Science, Faculty of Engineering and Information Technology, University of Technology Sydney, Sydney, NSW, Australia; ^2^Department of Civil Engineering and Computer Science Engineering, University of Rome Tor Vergata, Rome, Italy; ^3^Technogym, Cesena, Italy; ^4^Institute for Neural Computation, University of California, San Diego, La Jolla, CA, United States

**Keywords:** brain computer interface, cognitive neuroscience, machine learning, unification, reusability

## 1 Scope

Brain-computer interfaces (BCIs) enable people to interact with their environment (computers, robots, wheelchairs, etc.) by directly using brain signals, bypassing the natural pathways of nerves and muscles (Singh et al., 2021). Over the past decade, BCIs have shown tremendous progress, with increasing efforts to move beyond controlled laboratory environments to real-world settings. This highly interdisciplinary research field brings together experts from neuroscience, mathematics, computer science, engineering, and other disciplines. These researchers contribute to BCIs by developing neural understanding, new methods, techniques, BCI paradigms (Abiri et al., 2019), brain signal recording methods, acquisition tools/devices, and generating vast amounts of data.

Although most of these knowledge and information are regularly available through peer-reviewed platforms and shared via open-access data and method repositories such as MOABB (Jayaram and Barachant, 2018), still a significant challenge comes in the form of interpreting, reusing, comparing, and benchmarking these resources. This challenge is growing significantly between computational intelligence and neuroscience research due to the current flow of readily available tools and devices.

While many methods, datasets, and techniques are openly accessible for researchers to reuse, the multidisciplinary nature of BCI research creates significant gaps in sharing methods and datasets, comparing results, and reproducing experiments. This gap exists because researchers often share domain-specific information that can be difficult for those in other disciplines to interpret. Consider what would be needed to reproduce a steady-state visually evoked potential (SSVEP)-BCIs (Lin et al., 2018), besides sharing data. There is a need for information like the number of unique flickering stimuli presented to the user, the flickering rate, and signal processing specific details such as the impedance of electrodes, type of reference used, applied signal filters, appropriate labels, etc. These details are usually available in related publications but are hard to interpret for non-domain-specific researchers.

The research presented here alleviates these caveats by providing a novel approach for researchers from the multidisciplinary domain of BCI to share their advances in the interdisciplinary research field.

## 2 Research highlights

Developing a unified framework to address the need for more community standards in applying machine learning problems is one of the crucial problems. The work by Benerradi et al. introduced a novel framework called BenchNIRS, a benchmarking framework for functional near-infrared spectroscopy (fNIRS) data that aims to establish best practices for machine learning methodology, optimize models, and evaluate them without bias. This framework allows researchers to validate classification results across five open-access datasets using six baseline machine-learning models: linear discriminant analysis (LDA), support vector machine (SVM), k-nearest neighbors (kNN), artificial neural network (ANN), convolutional neural network (CNN), and long short-term memory (LSTM). Their findings revealed that while no single model consistently outperformed others across all datasets, the LDA model remained a robust and reliable choice despite its simplicity. This work contributes significantly to the fNIRS community by providing a structured approach to benchmarking machine learning models, offering practical recommendations for methodology decisions, and fostering a collaborative effort to establish community standards.

In addition, community standards in machine learning would be as good as the data provided, which is also required to have significant challenges. Bianchi et al. address the challenge by merging multiple BCI P300 speller datasets based on 127 subjects, encompassing more than 6,800 spelled characters and 1,168,230 stimuli into a unified format, emphasizing the perspectives and pitfalls of such an endeavor. The study demonstrates the necessity of standardizing data formats and repositories to facilitate data sharing and enhance the reproducibility of BCI research. They focused on datasets available in the public domain, aiming to create a standardized file format that encapsulates all necessary information for training and testing machine learning classifiers. They have developed a new format that includes detailed annotations, such as features, labels, and other essential metadata. This eliminates the need for additional documents or scripts for data and feature extraction. This work also highlights the importance of maintaining raw, unprocessed data, as this allows for consistent preprocessing and analysis across different studies. The standardized dataset format for P300 enhances current research capabilities but also sets a precedent for future data-sharing practices in the neurotechnology community.

A unified dataset and set machine learning evaluation standards can significantly improve data sharing and maximize the bias-free machine learning evaluation. However, this process can be further enhanced by understanding insights into the generalizability of neural features and applying them to develop machine learning algorithms. The article by Fox et al., as well as Singh and Bianchi, investigates and proposes such approaches where a machine learning method has been developed that can generalize over different BCI experimental conditions and sessions. In Fox et al., the author employed various approaches based on various neural features to train and test 20 SVM models derived from EEG data. The results vary due to non-stationarity and temporal variability in EEG signals, thus highlighting the nature of signals. The research underscores the complexity of developing BCIs and emphasizes the need for continuous machine learning model adaptation.

In a similar node of generalization of the machine learning method, Singh and Bianchi explored the encoding of temporal information in deep convolutional neural networks (CNNs) for EEG data. They have demonstrated that deep learning techniques could automate the process of EEG signal analysis, extracting crucial time-dependent features directly from raw data. This approach reduces the need for manual preprocessing and feature extraction, making it a promising direction for real-time BCI applications. The study's results indicated that incorporating both frequency and temporal information significantly improved the accuracy of EEG signal classification, paving the way for more robust BCI systems.

## 3 Summary

Developing and standardizing machine learning frameworks and data formats are crucial for advancing brain-computer interface (BCI) research. Benerradi et al.'s BenchNIRS framework and Bianchi et al.'s unified P300 dataset format address significant data sharing and reproducibility challenges, setting new precedents for the neurotechnology community. Meanwhile, Fox et al. and Singh and Bianchi highlight the importance of generalizing neural features and automating EEG analysis to enhance the accuracy and robustness of BCIs. These efforts collectively pave the way for more reliable, efficient, and widely applicable BCI systems, fostering greater collaboration and innovation.

## Author contributions

AS: Writing – review & editing, Writing – original draft. LB: Writing – review & editing. DV: Writing – review & editing. MN: Writing – review & editing.
